# Small cell neuroendocrine tumor of the urinary bladder: A case report

**DOI:** 10.1016/j.ijscr.2025.111045

**Published:** 2025-02-11

**Authors:** Divya Shetty, Adwaita Mashelkar, Anita Sharan, Sudhamani S.

**Affiliations:** Department of Pathology, Dr. D.Y. Patil School of medicine, Nerul, Navi Mumbai, Maharashtra, India

**Keywords:** Case report, Diagnosis, Multimodal treatment, TURBT, PUNLMP, Small cell neuroendocrine carcinoma, Bladder cancer

## Abstract

**Introduction and importance:**

Bladder cancer is predominantly characterized by urothelial carcinomas, but small cell neuroendocrine carcinomas (SCNEC) represent a rare and aggressive subset.

**Case presentation:**

A 50-year-old male presented with painless hematuria and voiding difficulties. Ultrasonography revealed a 3.2 × 3 × 1.8 cm lesion with irregular margins and microcalcifications in the bladder. Computed tomography urography suggested an intra-vesical protruding mass. The patient underwent transurethral resection of the bladder tumor (TURBT). Histopathological examination confirmed SCNEC and papillary urothelial neoplasm of low malignant potential (PUNLMP), with high Ki-67 (90 %). PET-CT ruled out metastasis. Treatment included chemotherapy followed by radiotherapy.

**Clinical discussion:**

At one-year follow-up, the patient was undergoing radiotherapy after completing chemotherapy. This case highlights the importance of multimodal treatment for aggressive bladder cancers, emphasizing TURBT, adjuvant chemotherapy, and radiotherapy**.**

**Conclusion:**

Comprehensive management, including chemotherapy and radiotherapy, is critical in treating rare and aggressive bladder cancers like SCNEC, leading to improved outcomes.

## Highlights

• Primary neuroendocrine carcinomas of the urinary bladder are rare; represent an aggressive form of cancer with a poor prognosis.
• Case presents an unusual coexistence of SCNEC and papillary urothelial neoplasm of low malignant potential.
• High Ki-67 index suggests the tumor's rapid growth and aggressive nature, necessitating an intensive treatment approach.
• Comprehensive treatment strategy;transurethral resection, chemotherapy, and radiotherapy, was employed to manage the dual lesion.
• Patient showed a positive response to treatment, with no lymph node involvement and radiotherapy at the one-year follow-up.

## 1 Introduction

Urinary bladder cancer is predominantly composed of urothelial carcinomas, which account for over 90 % of cases [1]. Neuroendocrine tumors, including small cell neuroendocrine carcinoma (SCNEC), are rare, making up <0.5 % of genitourinary cancers [1,2]. SCNEC is highly aggressive, with 5-year survival rates ranging from 8 % to 25 % [3]. This case report describes SCNEC coexisting with a papillary urothelial neoplasm of low malignant potential (PUNLMP) in a 50-year-old male patient. We aim to provide a comprehensive analysis, focusing on its clinical-pathological features.

While the standard treatment for SCNEC of the bladder is not well-defined, multimodal therapy, including neoadjuvant chemotherapy, transurethral resection, adjuvant chemotherapy, and radiotherapy, is preferred over surgery alone [4,5]. Chemoradiation is effective for controlling local-regional disease and can preserve bladder function, offering an alternative to cystectomy, even in advanced cases [6]. Patients treated with multimodal approaches have shown favorable outcomes, including prolonged survival without recurrence [5].

This work has been reported in line with the SCARE criteria [7].

## 2 Case presentation

A 50-year-old male presented to the outpatient department with a two-month history of painless hematuria and voiding difficulties. There is no notable past or family medical history. Patient denied any addiction. The physical examination showed signs of pallor, and the laboratory results revealed a hemoglobin level of 9.0 g/dL. Ultrasonography revealed a 3.2x3x1.8 cm well-defined hypoechoic lesion with irregular margins, specs of microcalcification and increased vascularity in the posterior wall of the urinary bladder. Further investigation through computed tomography intravenous urography indicated a 2.8 × 2.5 × 2 cm endophytic tissue involving the left posterior aspect of the urinary bladder, suggestive of a neoplastic etiology.

Following these findings, the patient underwent a transurtheral resection of the bladder tumor (TURBT), and biopsy samples were obtained from both superficial and deep tissues for histopathological examination. The histopathological analysis revealed neoplastic urothelial cells arranged in a papillary pattern with a fibrovascular core. The urothelial cells appeared monotonous with minimal nuclear enlargement and maintained polarity without crowding or discohesion.

**Fig. 1:**
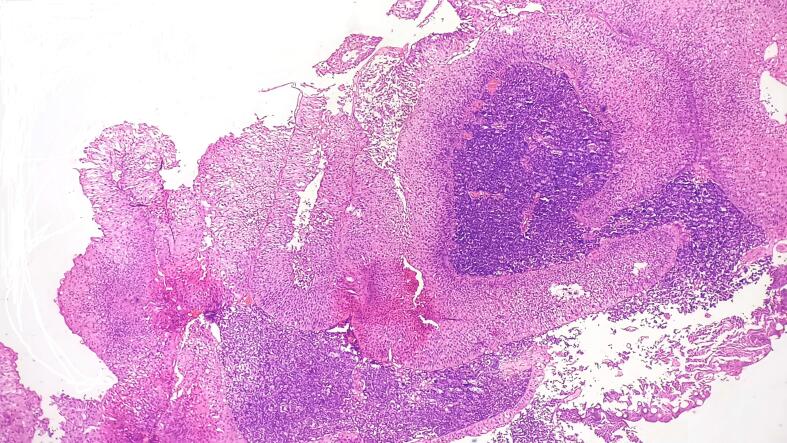
Neoplastic urothelial cells arranged in a papillary pattern with a fibrovascular core with maintained polarity lamina propria showing a small blue cell population (Hematoxylin and Eosin stain, 100×); (For interpretation of the references to colour in this figure legend, the reader is referred to the web version of this article.)

**Fig. 2:**
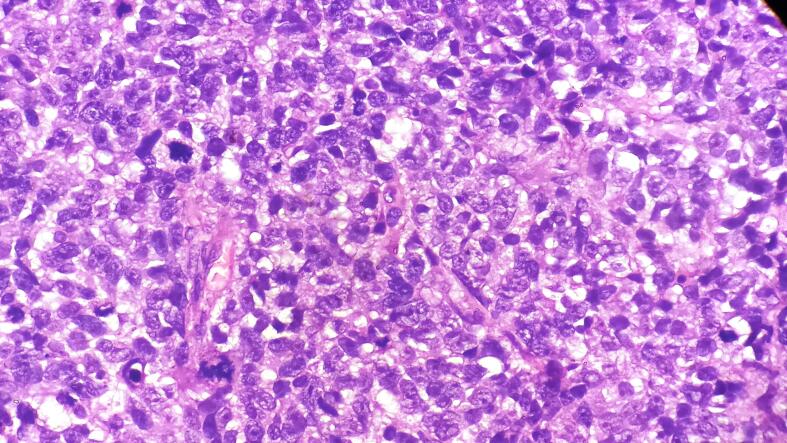
Sheets of small cells with minimal cytoplasm, nuclear molding, indistinct nucleoli, high mitotic activity, and apoptosis. (Hematoxylin and Eosin stain, 400×).

Interestingly within the lamina propria, a distinct tumor cell population was identified (Fig. 1). These cells exhibited characteristics of small cell carcinoma, including diffuse sheets of small cells with minimal cytoplasm, nuclear molding, indistinct nucleoli, high mitotic activity (10 to 12/hpf), and apoptosis (Fig. 2). Additionally, bizarre cells and tumor giant cells were observed, indicative of an aggressive tumor phenotype. The deeper biopsy also showed a similar tumor cell population which was also seen infiltrating into the detrusor muscle.

**Fig. 3:**
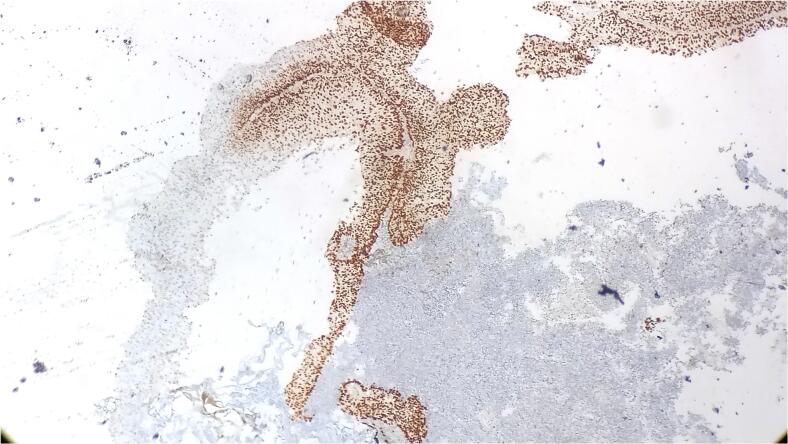
Strong and diffuse GATA 3 positivity in the superficially arranged papillary pattern of tumor cells but negativity in the small cell population.

**Fig. 4:**
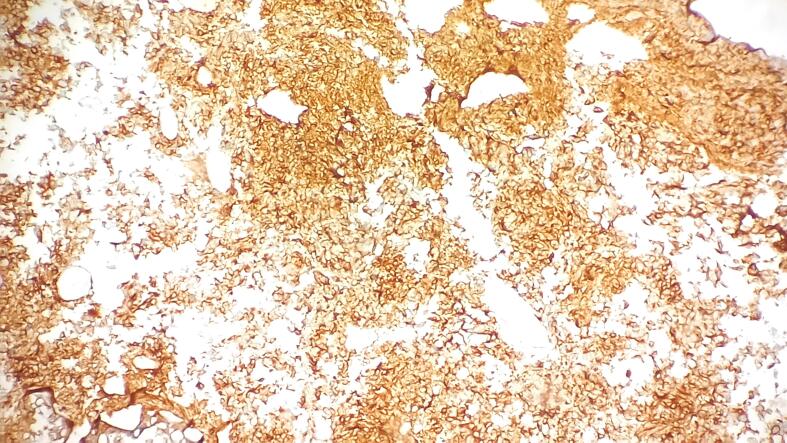
Strong and diffuse synaptophysin positivity in the small cell population.

**Fig. 5:**
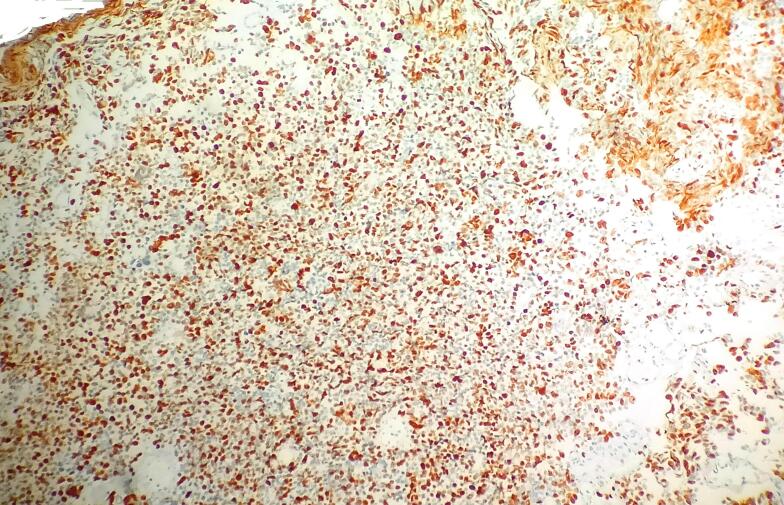
A high Ki 67 in the small cell population.

Immunohistochemistry further characterized the tumor, revealing strong and diffuse GATA 3 positivity in the superficially arranged papillary pattern of tumor cells but negativity in the small cell population (Fig. 3). Conversely, synaptophysin was positive in the small cell population but negative in the overlying papillary tumor cells (Fig. 4). Ki-67 proliferation index was notably high, around 90 % (Fig. 5).

Based on these findings, a diagnosis of SCNEC of the bladder (with invasion into the detrusor muscle) coexisting with papillary urothelial neoplasm of low malignant potential was established. A positron emission tomography-computed tomography was performed which ruled out metastasis to other sites.

The treatment plan involved initiating chemotherapy followed by radiotherapy to target the aggressive nature of the tumor and prevent further progression. At the one-year follow-up, the patient had completed the chemotherapy cycle and was undergoing radiotherapy.

## 3 Discussion

The comparative analysis of bladder cancer cases from various studies [8,9,10,14,15]. alongside the present case report reveals significant insights across several critical parameters: age, sex, clinical features, comorbidities, smoking status, tumor localization, lymph node metastasis, treatment strategies, diagnosis, Ki-67 proliferation index, and follow-up outcomes Table 1.

**Table 1:** Brief overview of studies relevant to SCNEC of bladder in the existing literature.

Author	Age	Sex	Clinical feature	Comorbidities	Smoking	Limited to bladder	LN metastasis	Treatment	Diagnosis	Ki-67 (%)	Follow-up (Mo)
Li et al., 2018 [8]	87	Male	Hematuria	No	Yes	No	No	Patient refused treatment	SCNEC	–	Dead (2) (non-compliant to treatment)
Oliveri et al., 2020 [9]	78	Male	Hematuria	Hypertension, dyslipidemia, chronic renal failure	Yes	Yes	No	TURBT, patient refused follow-up cystectomy. CTx and RTx was contraindicated	HGUC + SCNEC	>20	Dead (6) (metastasis)
Masood et al., 2020 [14]	60	Male	Hematuria, dysuria	No	–	Yes	No	TURBT + CTx	SCNEC	>20	Alive (13)
Eshraghi et al., 2022 [15]	50	Male	Hematuria	No	Yes	No	Yes	Radical cystprostectomy + CTx + RTx	SCNEC	80	Alive (12)
Sarver et al., 2022 [10]	70	Female	Hematuria	Hypertension, hypothyroidism, chronic kidney disease	Yes	Yes	Yes	TURBT + CTx	SCNEC with squamous differentation	>20	Alive
Present study	50	Male	Hematuria, dysuria	No	No	Yes	No	TURBT + CTx + RTx	PUNLMP + SCNEC	>90	Alive (15)

(CTx = chemotherapy, HGUC = high grade urothelial carcinoma, LN = lymph node, Mo = months; PUNLMP = papillary urothelial neoplasm of low malignant potential, RTx = radiotherapy, SCNEC = small cell neuroendocrine carcinoma, TURBT = transurethral resection of bladder tumor).

Age is a crucial determinant in bladder cancer outcomes. The present case report features a 50-year-old male, contrasting sharply with older cohorts such as those in Li et al. [8] (87 years) and Oliveri et al. [9] (78 years). The younger patient in the current case report likely experienced better overall health and treatment tolerance, facilitating a more aggressive therapeutic approach. Older patients in previous studies often faced significant health issues that limited treatment options, contributing to poorer prognoses. For instance, Li et al.'s [8] patient refused treatment, leading to death within two months, underscoring how age-related health challenges can complicate cancer management.

All studies included predominantly male patients, aligning with the trend that bladder cancer is more prevalent in men. The present case report's male patient reflects this demographic, while Sarver et al. (2022) [10] included a female patient. This highlights the gender disparity in bladder cancer incidence and suggests a need for targeted awareness and screening initiatives focused on men, although the presence of female cases should not be overlooked.

Clinical presentation was largely consistent across studies, with hematuria reported as the primary symptom. The present case report noted additional dysuria, indicating possible disease progression. Such clinical features stress the importance of prompt evaluation and diagnosis in patients presenting with urinary symptoms. The presence of both hematuria and dysuria in the current case suggests a more complex clinical scenario, warranting comprehensive diagnostic assessments. Physicians need to be vigilant, maintaining a high index of clinical suspicion for prompt and effective treatment, even in cases of patient-reported symptoms. Endoscopic diagnosis should not be delayed, even in cases of negative lab and imaging workups, as a lesion may be hidden. A diagnostic challenge can also arise, even in cases with atypical symptoms, which necessitate further workup [11].

Comorbidities significantly influenced treatment options and outcomes across studies. In cases like Oliveri et al. [9] and Sarver et al. [10], patients presented with various comorbidities, including hypertension and chronic kidney disease, which complicated their clinical management and contributed to poorer survival rates. Conversely, the present case report's patient had no notable comorbidities, allowing for an aggressive treatment plan. This lack of comorbidities likely enhanced the patient's ability to tolerate intensive therapies, underscoring the importance of evaluating comorbid conditions in treatment planning.

Smoking is a well-established risk factor for bladder cancer. In earlier studies, several patients were smokers, which likely contributed to more aggressive disease presentations and poorer outcomes. The present case report's non-smoking patient contrasts with those in prior studies, where smoking was prevalent. This suggests that non-smokers may experience fewer complications and better responses to treatment.

Tumor localization plays a critical role in determining prognosis. The present case report reported a tumor limited to the bladder without lymph node involvement, which is associated with better outcomes. In contrast, previous studies noted cases where tumors were more advanced, either extending beyond the bladder or involving lymph nodes. For instance, Sarver et al. [10] reported lymph node involvement, which typically correlates with worse prognoses. This finding underscores the importance of early diagnosis and localized disease management in improving survival rates.

Lymph node involvement is a significant prognostic factor in bladder cancer. In previous studies, such as those by Oliveri et al. [9] and Sarver et al. [10], patients with lymph node metastasis faced more complex treatment decisions and worse outcomes. The present case report, however, indicates no lymph node involvement, likely contributing to the successful treatment outcome. This reinforces the importance of localized disease in achieving better survival rates, emphasizing the need for prompt intervention in early-stage bladder cancer.

Treatment approaches varied widely across the studies. The present case report employed a comprehensive trimodality strategy involving TURBT, chemotherapy, and radiotherapy, reflecting a more aggressive and multimodal approach. In contrast, earlier studies, particularly Li et al. [8], reported patients who refused treatment or had limited intervention, leading to poorer outcomes. For example, the patient in Li et al. [8] died shortly after refusing treatment. This highlights the critical role of timely and aggressive treatment in improving survival outcomes, especially in younger patients without significant comorbidities.

In addition to standard treatments, emerging therapies like immunotherapy have shown promise in managing neuroendocrine tumors, though their role in SCNEC remains under investigation**.** Immunotherapy, including immune checkpoint inhibitors, has been explored in treating various bladder cancers and may offer additional options for patients with recurrent or metastatic disease [9]. BCG therapy is generally used in urothelial carcinoma, but its role in SCNEC, especially in cases with concurrent urothelial features, is still under exploration [10]**.**

En bloc resection of bladder tumors (ERBT) is a promising alternative to standard transurethral resection of bladder tumors (TURBT) for non-muscle invasive bladder cancer. ERBT offers improved staging, higher detrusor muscle detection, and may reduce the need for re-TURBT. Although safe and effective, its adoption remains limited due to certain challenges in implementation [12,13].

While multimodal treatments offer significant benefits, they are not without complications. The combination of chemotherapy, radiation, and immunotherapies can lead to adverse effects such as bladder rupture, radiation cystitis, and immune-related side effects**.** Recent studies have documented such complications, highlighting the importance of patient monitoring during treatment [[4], [5], [6]]. It is crucial to carefully weigh the risks and benefits of aggressive treatment strategies and adjust treatment protocols to minimize the potential for severe side effects**.**

The diagnosis in the present case report included PUNLMP and SCNEC, with a Ki-67 index exceeding 90 %, indicating a highly proliferative tumor. This is in contrast to previous studies, where Ki-67 levels ranged from >20 % to 80 %, suggesting varying degrees of tumor aggression. The high Ki-67 level in the current case indicates a more aggressive tumor, but the effective treatment strategy led to a favorable outcome, with the patient alive at 15 months.

Surveillance for neuroendocrine tumors, particularly SCNEC of the bladder, is crucial. Regular imaging and monitoring of tumor markers like Ki-67 are essential to detect recurrence and manage long-term outcomes**.** Early intervention is key to improving survival rates and reducing the risk of metastasis. Following multimodal treatment, careful follow-up ensures early detection of recurrence and addresses any complications that may arise from the aggressive treatment regimen**.**

## 4 Conclusion

In conclusion, the comparative analysis of bladder cancer cases across the present case report and previous literature highlights critical factors influencing patient outcomes. Age, comorbidities, smoking status, tumor localization, lymph node metastasis, treatment strategies, and Ki-67 levels all play significant roles in determining prognosis. The present case report underscores the potential benefits of multimodal treatment in managing aggressive bladder cancers like SCNEC, emphasizing its superiority over surgical therapy alone. The combination of chemotherapy, radiotherapy, and potentially newer therapies such as immunotherapy offers improved survival rates and better long-term outcomes compared to surgery alone.

## CRediT authorship contribution statement

All authors have contributed towards the conception and development, data collection, data analysis and interpretation, preparation and critical revision of this manuscript.

## Consent

Written informed consent was obtained from the patient for publication and any accompanying images. A copy of the written consent is available for review by the Editor-in-Chief of this journal on request.

## Ethics approval

Ethics committee approval was not required for this case report, as the patient's information was anonymized, and no identifiable data are included. The case was reviewed and anonymized in accordance with institutional guidelines on the use of medical records for educational purposes.

## Guarantor

Name: Dr. Divya Shetty

Email: divya.shetty@dypatil.edu

Phone: +919004148824

## Source of funding

This case report was not supported by any funding.

## Registration of research studies

1.Name of the registry: Not applicable.

2.Unique identifying number or registration ID: Not applicable.

3.Hyperlink to your specific registration (must be publicly accessible and will be checked): Not applicable.

## Declaration of competing interest

The authors declare that they have no conflict of interest.
